# Larvæ of the Common Fly in the Meatus Auditorius Externus

**Published:** 1862-07

**Authors:** John Ellis Blake

**Affiliations:** Middletown, Conn.


### SELECTIONS.

---

*Larvæ of the Common Fly in the Meatus Auditorius Externus. By* JOHN ELLIS BLAKE, M. D., *of Middletown, Conn.*

On the 21st of August, 18—, a young man from a neighboring town, accompanied by his parents, presented himself at the office, complaining of a most severe pain in the ear. This pain was somewhat paroxysmal in its character, and was accompanied by a discharge of arterial blood. His parents represented that he had always been perfectly well until the 17th of the month, when the pain and discharge of blood first began; and the amount lost they estimated at nearly a pint. Making due allowance for common over-estimates of hæmorrhage, I can readily believe that he had lost a dozen ounces. In the course of conversation with them, something was said of "worms" as being the cause of his distress; but having to encounter the *vermicular* theory of disease at every turn in country practice, I paid little attention to the remark then. In endeavoring to get some light as to the previous history of the case, I found the young man himself referred his troubles to "going in bathing" some days previous to the 17th; after which bath, he laid down and slept for a time on the grass. On wakihg, he felt something in his ear, and supposed it to be a "bubble," but could not get rid of it. I have spoken of the young man's distress as *pain*. The word can hardly convey an idea of the agony he seemed to be in, every few seconds. The perspiration poured from his forehead, and clasping his head with his hands, he would roll upon the floor, seemingly almost delirious with his sufferings. With difficulty, owing to the flow of blood and the involuntary movements of the patient, I was able to introduce a speculum into the meatus, and get a glimpse of the interior. What was my astonishment to find it tenanted by several maggots, in full activity! The movements of these creatures against the sensitive structures of the ear was evidently the cause of the pain, and their excavations in feeding the cause of the hæmorrhage. I found that the least attempt to remove them by instruments was resisted by them; and as they were immediately set in motion thereby, wriggling against the membrana tympani, the agony caused the patient was too much for human endurance, and he could not be held in the chair. I consequently desisted from such attempts, after a fair trial of various instruments. Remembering some experiments I once saw tried upon these larvæ, tending to show their extreme tenacity of life for a time, even when immersed in caustic substances, I wasted no time, after a few trials with syringing, but, catching sight of a conical ether sponge upon the table, it occurred to me that anæsthesia might do. The sponge was wet with sulphuric ether and held for a few seconds over the ear, when we had the satisfaction of seeing the maggots wriggle from the ear, in great haste, on removing it. On looking in, but one could be seen, and a re-application of the sponge soon caused him to come out for fresh air. The whole number removed was some eight or ten; and, as the larvæ were of good size, I can hardly see how they could have been packed away so deeply in the passage, but such was the case. I fed them carefully until they became perfect insects, and found them, as I had supposed would be the case, the small, green-bellied fly, commonly called the "blow-fly," and familiar to all.

I would say that the ear thus affected was a perfectly healthy one, and having no unnatual secretion.—*Boston Medical Journal,*

